# Using multi-omics to explore the effect of *Bacillus velezensis* SAAS-63 on resisting nutrient stress in lettuce

**DOI:** 10.1007/s00253-024-13153-y

**Published:** 2024-04-29

**Authors:** Yinshuang Bai, Ke Song, Mengxiang Gao, Juan Ma, Yifan Zhou, Hua Liu, Haijuan Zeng, Jinbin Wang, Xianqing Zheng

**Affiliations:** 1 College of Life Sciences, Yangtze University, Jingzhou, 434025 China; 2 Key Laboratory of Agricultural Genetics and Breeding, The Biotechnology Research Institute, The Eco-Environmental Protection Research Institute, Shanghai Academy of Agricultural Sciences, Shanghai, 201106 China; 3 Crops Ecological Environment Security Inspection and Supervision Center, Key Laboratory for Safety Assessment of Agricultural Genetically Modified Organisms, Ministry of Agriculture and Rural Affairs, Shanghai, 201106 China; 4 School of Life Science and Engineering, Lanzhou University of Technology, Lanzhou, 730050 China

**Keywords:** Nutrient stress, PGPR, Microbial community structure, Microbial diversity, Metabolites, Correlation

## Abstract

**Abstract:**

To avoid the unreasonable use of chemical fertilizer, an environmentally friendly means of improving soil fertility is required. This study explored the role of the plant growth-promoting rhizosphere bacteria (PGPR) strain *Bacillus velezensis* SAAS-63 in improving nutrient stress in lettuce. Compared with no inoculation, *B. velezensis* SAAS-63 inoculants exhibited significantly increased fresh weight, root length, and shoot height under nutrient deficiency, as well as improved antioxidant activities and proline contents. The exogenous addition of *B. velezensis* SAAS-63 also significantly increased the accumulation of macroelements and micronutrients in lettuce. To elucidate the resistance mechanisms induced by *B. velezensis* SAAS-63 under nutrient stress, high-throughput sequencing and multi-omics analysis were performed. Inoculation with *B. velezensis* SAAS-63 altered the microbial community of the rhizosphere and increased the relative abundances of *Streptomyces*, *Actinoallomurus*, *Verrucomicrobia*, and *Chloroflexi.* It is worth noting that the inoculant SAAS-63 can affect plant rhizosphere metabolism. The inoculant changed the metabolic flow of phenylpropanoid metabolic pathway under nutrient deficiency and promoted phenylalanine to participate more in the synthesis of lignin precursors and coumarin substances by inhibiting the synthesis of flavone and isoflavone, thus improving plant resistance. This study showed that the addition of inoculant SAAS-63 could help plants recruit microorganisms to decompose and utilize trehalose and re-established the carbon metabolism of the plant rhizosphere. Additionally, microbes were found to be closely related to the accumulation of metabolites based on correlation analysis. The results indicated that the addition of PGPRs has an important role in regulating soil rhizosphere microbes and metabolism, providing valuable information for understanding how PGPRs affect complex biological processes and enhance plant adaptation to nutrient deficiency.

**Key points:**

*• Inoculation with SAAS-63 significantly promoted plant growth under nutrient-deficient conditions*

*• Inoculation with SAAS-63 affected rhizosphere microbial diversity and community structure*

*• Inoculation with SAAS-63 affected plant rhizosphere metabolism and induced plants to synthesize substances that resist stress*

**Graphical abstract:**

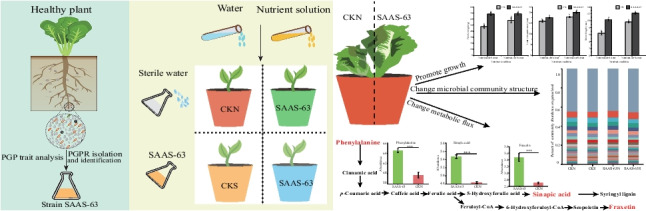

**Supplementary Information:**

The online version contains supplementary material available at 10.1007/s00253-024-13153-y.

## Introduction

As an abiotic stress, nutritional stress seriously influences plant growth and yield. Currently, the loss, imbalance, and shortage of soil nutrients caused by unsustainable farming systems are becoming increasingly extensive and are not conducive to the improvement of crop yield. Plant growth and development require adequate and balanced mineral nutrition. There are a variety of mineral nutrients in soil, which affect several cellular and metabolic processes (Gupta et al. 2017). In addition, a deficiency in mineral elements has seriously restricted the growth of crops around the world (Haefele et al. 2014). Macroelements and microelements have positive effects on plant growth and development. Deficiencies in nitrogen (N) and phosphorus (P) are considered to be the most serious causes of loss of agricultural production globally (Rose et al. 2016). Nitrogen deficiency can lead to the decrease of chlorophyll content, inhibit photosynthesis, and reduce dry matter accumulation in plants (Du et al. 2022). Phosphorous deficiency reduces photosynthetic capacity and darkens the plant leaves (Sun et al. 2021). Nutrient deficiency is a complex problem, and plants often lack not just one element but multiple elements. Therefore, to resolve nutrient deficiency, the use of chemical fertilizers has increased dramatically, leading to soil health and environmental problems.

More and more studies have revealed the potential of microorganisms in agriculture (Liu et al. 2023). Plant growth-promoting rhizosphere bacteria (PGPRs) are considered to be an effective method for maintaining agricultural productivity and promoting the sustainable development of agriculture. A previous study showed that PGPRs play an great role in improving plants resistance to abiotic stress (Gupta et al. 2022), such as by inducing the antioxidant system and increasing the activity of antioxidant enzymes and the accumulation of osmotic regulatory substances (El-Esawi et al. 2019). Furthermore, PGPRs has the ability to maintain water balance, dissolve phosphorus and potassium, fix nitrogen, and chelate iron in plants (Santoyo et al. 2016). In addition, PGPR also plays a key role in regulating plant rhizosphere microbial communities. Recently, studies have shown that PGPRs can alter the soil rhizosphere microbial community. For example, inoculation of two kinds of PGPR altered the microbial community of the rhizosphere soil of *Taxus chinensis* var. *mairei* (Bai et al. 2020). Moreover, studies have shown that several PGPRs can promote plant growth and improve plant tolerance to stress through multiple complex mechanisms (Shameer and Prasad 2018).

Metagenomic analysis has enabled a deeper understanding of the classification and functional diversity of soil microbial communities (Du et al. 2023). Metagenomic sequencing has been used to identify microorganisms that are differentially abundant in soils compared with soils not inoculated with PGPRs. Metabonomics provides detailed metabolite map information and can identify metabolic pathways of different concentrates, which helps to reveal the mechanism of interaction between microbial communities and the environment (Liu et al. 2017). Previously, many nontargeted metabolomics studies have been conducted in many crops to explain the mechanisms of tolerance to different stresses (Zhao et al. 2021). Currently, our understanding of the changes in the rhizosphere microbial community and rhizosphere metabolites of plants inoculated with PGPRs under nutrient deficiency is very limited. Therefore, to further understand the mechanism by which PGPRs improve plant resistance, metagenomic and metabolomic analyses are needed in addition to the determination of physiological and biochemical indices.

In this experiment, lettuce was used as the research material, and the PGPR strain *Bacillus velezensis* SAAS-63 was used as the inoculant. Different concentrations of nutrient solution were used to establish nutrient-deficient and nutrition-sufficient conditions for pot experiments. The objectives of this study included (1) to explore the effects of this strain on lettuce growth under nutritional stress, (2) to measure the capacity of this strain on the reactive oxygen species (ROS) scavenging and osmotic regulation in lettuce under nutritional stress, (3) to explore the effects of strain SAAS-63 inoculation on nutrient accumulation in lettuce, and (4) to reveal the mechanism by which *B. velezensis* SAAS-63 helps plants resist nutritional stress from multiple perspectives.

## Materials and methods

### Biological material and plant materials

Strain SAAS-63 is a PGPR isolated and screened from the plant rhizosphere. It was identified as *B. velezensis*. The strain SAAS-63 is preserved at Guangdong Microbial Culture Collection Center and the preservation number is GDMCC No: 63201. Strain *B. velezensis* SAAS-63 was used for inoculation in liquid Luria–Bertani (LB) medium. In order to avoid the influence of irrelevant factors such as LB medium on the experimental results, in this study, the cultured bacterial solution was centrifuged and added to sterile water to prepare bacterial suspension.

The lettuce (*Lactuca sativa* Linn) seeds used in this experiment obtained from the Biotechnology Research Institute Shanghai Academy of Agricultural Sciences, China. The seeds were surface-sterilized and washed with sterile distilled water. The seeds were germinated until they grow to the three-leaf stage. Then, we transplanted them into pots for culture. The control conditions of the artificial climate chamber were as follows: temperature controlled at 25 °C, relative humidity maintained at 60%, and 14 h/10 h light/dark cycle.

### Nutrient stress treatments and experimental design

Seedlings were transferred and cultured into pots containing vermiculite and saturated with complete nutrients for the nutrient-sufficient treatment and with water for the nutrient-deficient treatment. The nutrient solution was prepared according to Bisht’s method (Bisht et al. 2020). The original soil had pH 6.81, nitrogen 38.4 mg/kg, phosphorus 1.6 mg/kg, potassium 45.1 mg/kg, calcium 178.5 mg/kg, manganese 1.8 mg/kg, and zinc 0.4 mg/kg, all of which were at the deficiency level.

This experimental treatment was based on a completely randomized design and carried out 10 biological replicates. The two factors of experimental treatment were as follows: (1) the first factor consisted of two nutrient condition treatments, namely, (i) nutrient-deficient condition and (ii) nutrient-sufficient condition. (2) The second factor included two bacterial inoculation treatments, namely, (i) inoculated *B. velezensis* SAAS-63 and (ii) non-inoculated *B. velezensis* SAAS-63. The lettuce at the three-leaf stage was treated experimentally. When lettuce grew to the three-leaf stage, nutrition treatment (50 mL) and bacterial treatment (50 mL) were carried out every week for 4 weeks. Bacterial treatment began after three days of nutrition treatment. Samples were collected 5 days after the last inoculation.

### Morpho-physiological parameters and biochemical index measurement

The samples were harvested for 30 days after the first treatment. The fresh weight, leaf circumference, shoot length, and other main characters of lettuce were determined after the end of the experiment. Ten plants were randomly selected for each treatment. The total soluble sugars (SS) in the plants were determined following the anthrone method of Fales (1951). The absorbance was measured at 630 nm. Antioxidant enzymes such as superoxide dismutase (SOD), peroxidase (POD), and catalase (CAT) activities were measured using assay kits (Solarbio, Beijing, China). The proline (Pro) and malondialdehyde (MDA) contents were also determined using assay kits (Solarbio, Beijing, China). All measurements were performed according to the manufacturer’s instructions.

### Plant nutrient analysis

Total N in the lettuce samples were measured by the Kjeldahl method (Li et al. 2006). For elemental analysis (P, K, Ca, Mn, and Zn), each treated dry tissue sample was digested with HNO_3_. After the sample was digested, the element content was determined by inductively coupled plasma-emission spectrometry and inductively coupled plasma-mass spectrometry.

### Soil sample collection

The lettuce plants were removed from the pots after the bacterial and nutrient stress treatments. Large pieces of soil around the lettuce roots were removed and the lettuce rhizosphere carefully removed with a sterile brush. Plants rhizosphere soil samples were collected from the approximately 1-mm-thick soil layer attached to the roots of plants. Three pots were randomly selected from ten replicates, and the rhizosphere soil was passed through an 80-mesh sieve and mixed into a bulk sample. Finally, the collected soil samples were stored in 2-mL test tubes and preserved at – 80 °C for metagenomic and metabolomic analyses (three replicates) at Shanghai Majorbio Bio-Pharm Technology Co. Ltd. Shanghai, China.

### DNA extraction, Illumina NovaSeq sequencing, and data analysis

Genomic DNA was extracted from soil samples, and then, its concentration was tested using agarose gel electrophoresis. The V3-V4 region of the bacterial 16 s rRNA gene was amplified by 341F and 806R primers, respectively (Imparato et al. 2016). The ITS1-5F regions of the fungal ITS genes were amplified using the respective primer pairs ITS5-1737F and ITS1-2043R (Zhang et al. 2018). Sequencing libraries were constructed using the NEXTFLEX™ Rapid DNA-Seq Kit (Waltham, MA, USA). Sequencing was performed using the NovaSeq platform at Majorbio Bio-Pharm Technology Co. Ltd. (Shanghai, China). The soil microbial community dataset has been deposited into the NCBI Sequence Read Archive under accession number PRJNA1039622.

CD-HIT software (http://www.bioinformatics.org/cd-hit/) was used to cluster and construct a non-redundant gene set to explore the commonalities and differences between different samples (Fu et al. 2012). SOAPaligner (http://soap.genomics.org.cn/) was used to compare the high-quality reads for each sample with the non-redundant gene set software and to calculate the gene abundance information in the corresponding sample (Li et al. 2009). The alpha diversity index (Simpson) was calculated to assess microbial diversity. Principal component analysis (PCA) was used for rhizosphere microbial composition analysis and differential metabolite analysis. PCA analysis in metagenome was performed using the *ropls* package in R (https://rdrr.io/bioc/ropls/man/ropls-package.html).

### Rhizosphere soil metabolite analysis

The rhizosphere soil samples of no inoculation under nutrient-deficient condition (CKN), inoculation under nutrient-deficient condition (SAAS-63N), no inoculation under nutrient-sufficient condition (CKS), and inoculation under nutrient-sufficient condition (SAAS-63S) were frozen and stored in 1-mL Eppendorf tubes. Sample preparation for the metabonomic analysis was performed at Majorbio Bio-Pharm Technology Co. Ltd. (Shanghai, China) using standard procedures. The analytical system of UHPLC-MS/MS consists of ultra-high-performance liquid chromatography (UHPLC) system (Thermo Fisher, Waltham, MA, USA) and Q-accurate HF-X Fourier transform mass spectrometer. Raw data files generated by UHPLC-MS/MS were processed using the ProgenesisQI (Waters Corporation, Milford, MA, USA) for baseline filtering, peak identification, integration, peak alignment, and metabolite quantitation. Metabolites were annotated using the Kyoto Encyclopedia of Genes and Genomes (KEGG) database (Ogata et al. 1999) and the human metabolome database (HMDB database, https://hmdb.ca/). PCA analysis in metabonomics was performed using the *stats* package in R (https://rdrr.io/r/stats/stats-package.html). Differentially accumulated metabolites (DAMs) were distinguished using PCA and orthogonal projections to latent structures-discriminant analysis (OPLS-DA) model. The DAMs were screened according to fold change ≥ 2. Finally, the KEGG database was used for the pathway enrichment analysis of DAMs. The metabolite volcano plot was generated using the *ggplot2* package in R (https://rdrr.io/cran/ggplot2/).

### Statistical analysis

The significance of physiological and biochemical indexes of different treatments of lettuce was calculated by SPSS 26.0 (IBM Corp., Armonk, NY, USA). Differences in all treatments were assessed for significance using one-way analysis of variance (ANOVA). Figures were generated using Origin 2021 software (Origin Lab, Northampton, MA, USA). Statistically significant differences were defined at *p* < 0.05.

## Results

### Growth-promoting effect of *B. velezensis* SAAS-63 under nutrient deficiency

**Fig. 1:**
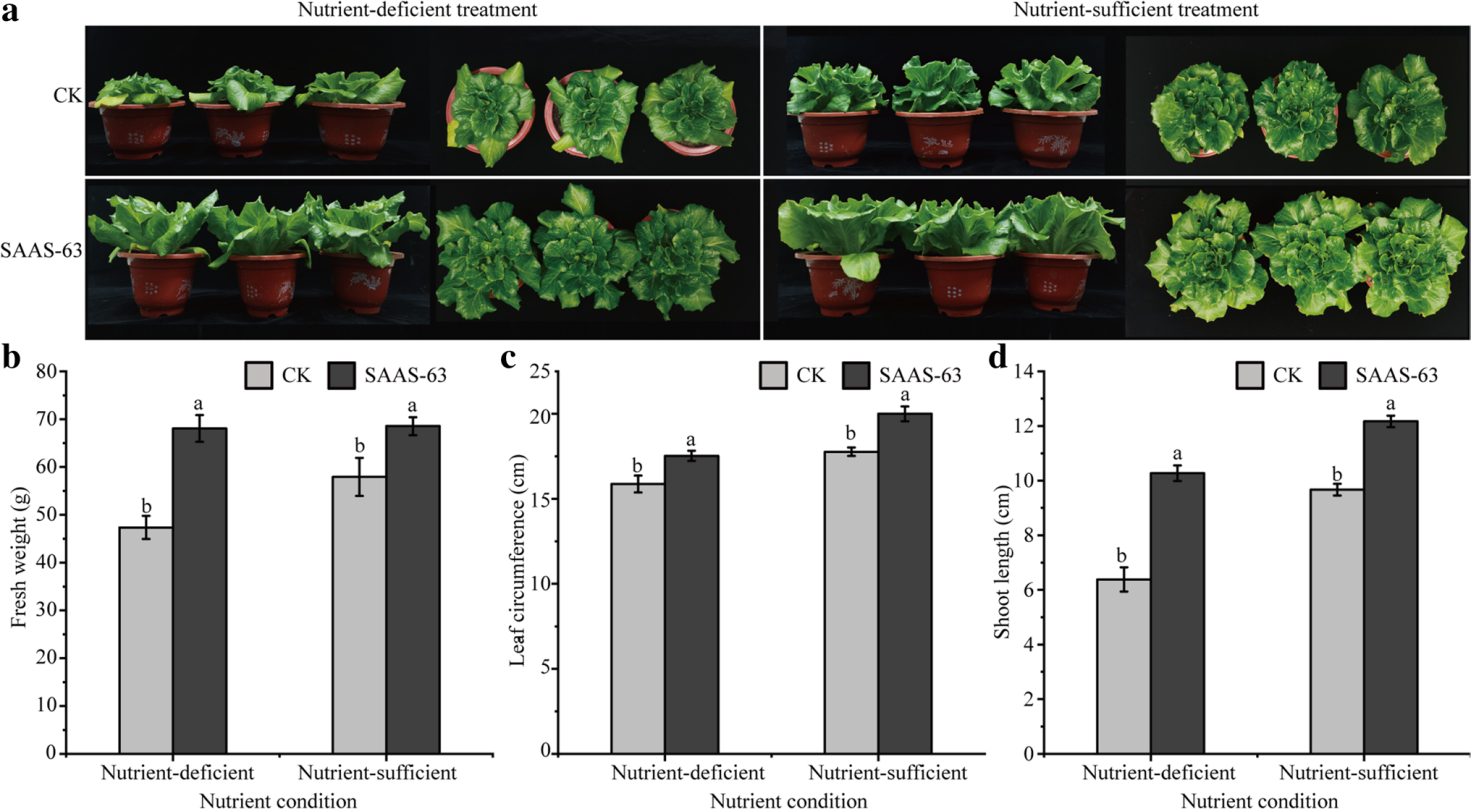
Effect of inoculation with *B. velezensis* SAAS-63 under different nutrient conditions on the **a** growth state of lettuce, **b** fresh weight, **c** leaf circumference, and **d** shoot length of lettuce. Data are means ± SD of three replicate samples. Values labeled with different letters are significantly different based on Tukey’s multiple range tests (*p* < 0.05). CK represents the control group not inoculated with SAAS-63, and SAAS-63 represents the treatment group inoculated with SAAS-63

Under nutrient-deficient conditions, the lettuce plants were small and their leaves grew slowly, while under nutrient-sufficient conditions, the plants grew better. After inoculation with strain SAAS-63, lettuce growth was significantly promoted under both nutritional conditions (Fig. 1a). Under nutrient-deficient conditions, the fresh weight, leaf circumference, and shoot length of the lettuce inoculated with strain SAAS-63 increased by 43.69%, 10.45%, and 60.97%, respectively. Under nutrient-sufficient conditions, the fresh weight, leaf circumference, and shoot length of the lettuce inoculated with strain SAAS-63 increased by 18.32%, 12.55%, and 25.85%, respectively (Fig. 1b–d). The results indicate that the inoculated strain SAAS-63 can promote plant growth under either condition, but especially under nutrient-deficient conditions, and inoculation with *B. velezensis* SAAS-63 can improve plant growth.

### Effect of *B. velezensis* SAAS-63 on phytochemicals in lettuce

**Fig. 2:**
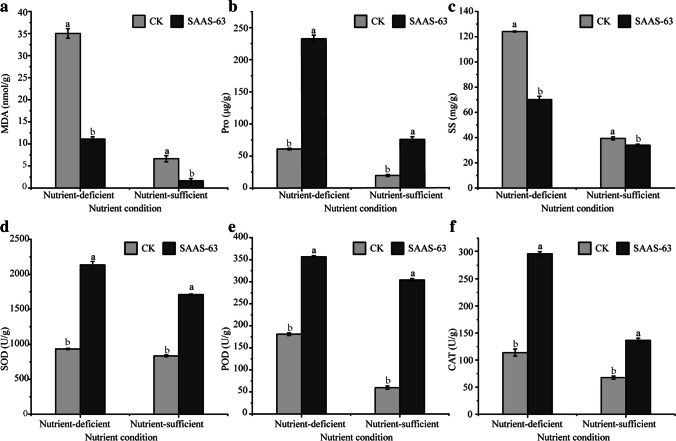
Effect of inoculation with *B. velezensis* SAAS-63 under different nutrient conditions on the **a** malondialdehyde (MDA) content, **b** proline (Pro) content, **c** soluble protein (SS) content, **d** superoxide dismutase (SOD) activity, **e** peroxidase (POD) activity, and **f** catalase (CAT) activity of lettuce. CK represents the control group not inoculated with SAAS-63, and SAAS-63 represents the treatment group inoculated with SAAS-63

Nutrient deficiency leads to plant dysplasia and inhibits the normal function of plants. We conducted a series of experiments to determine the effects of nutrient stress and PGPR inoculation on lettuce. The plants produced a large amount of MDA under nutrient deficiency but produced a small amount of MDA when nutrients were sufficient (Fig. 2a). The MDA content of plants under the two nutrient conditions inoculated with *B. velezensis* SAAS-63 decreased by 68.35% and 75.30% compared with CK. Under the two nutrient conditions, the Pro content of the plants after inoculation with *B. velezensis* SAAS-63 increased by 281.99% and 291.43% compared with CK (Fig. 2b). The SS content in the plants under nutrient-sufficient conditions was significantly lower than that under nutrient-deficient conditions, and the SS content in the plants under the two conditions was reduced by 43.43% and 13.77% following inoculation with *B. velezensis* SAAS-63 (Fig. 2c). Under different nutrient conditions, the activity of antioxidant enzymes in the plants differed. Compared with nutrient-sufficient conditions, the activity of antioxidant enzymes in the plants increased under nutrient deficiency, and inoculation with *B. velezensis* SAAS-63 greatly improved the activity of antioxidant enzymes in the plants under nutrient-deficient conditions. Under nutrient-deficient conditions, the activities of SOD, POD, and CAT increased by 56.26%, 97.06%, and 160.21%, respectively, after inoculation with *B. velezensis* SAAS-63 (Fig. 2d–f).

### Effect of *B. velezensis* SAAS-63 on nutrient accumulation in plant roots and leaves

**Fig. 3:**
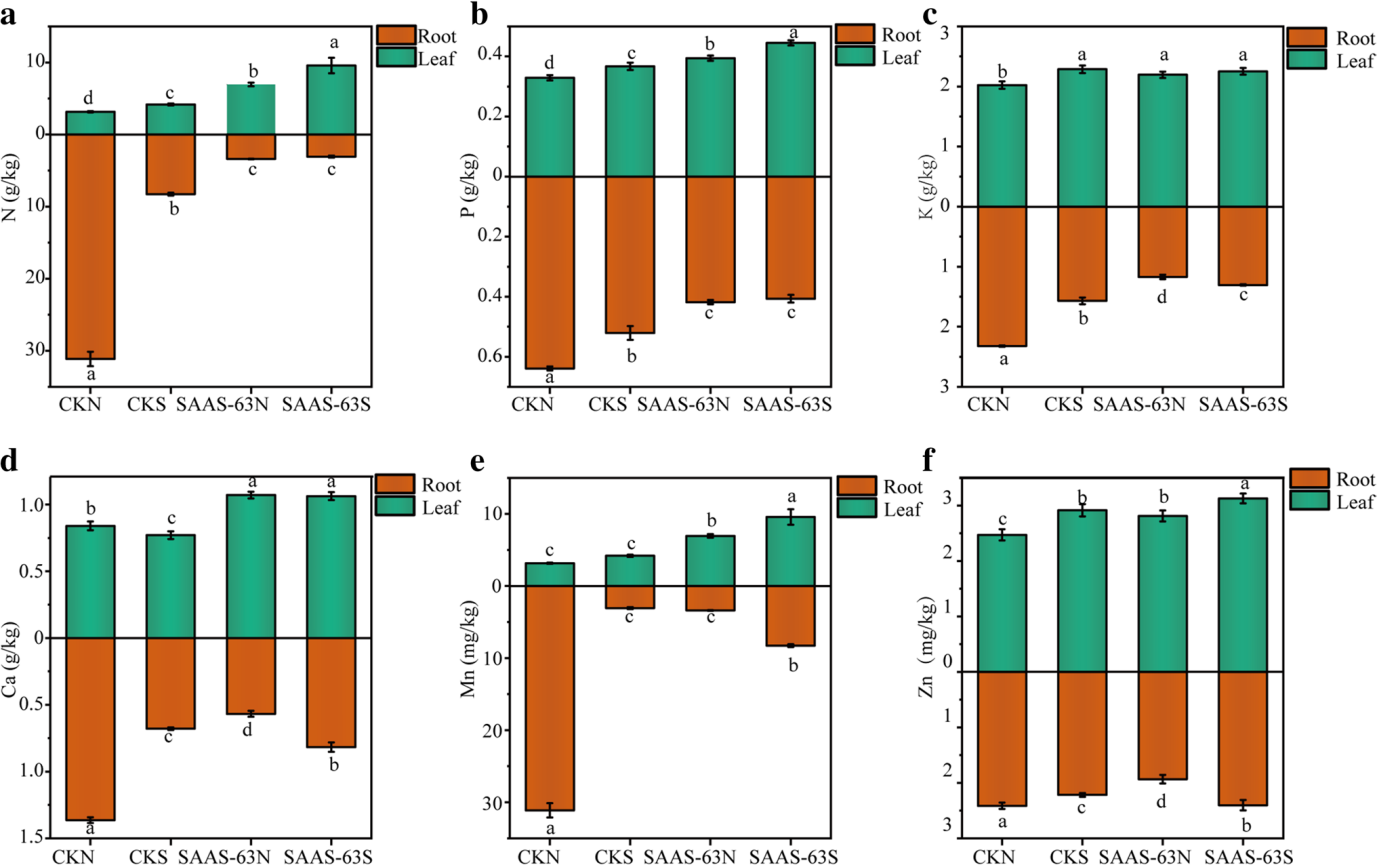
Effect of inoculation with *B. velezensis* SAAS-63 under different nutrient conditions on the contents of **a**–**c** macronutrients and **d**–**f** micronutrients. CKN and CKS represent the control groups without inoculation under the conditions of nutrient deficiency and nutrient sufficiency, respectively. SAAS-63N and SAAS-63S represent the groups that were inoculated with *B. velezensis* SAAS-63 under nutrient deficiency and nutrient sufficiency, respectively

To explore the effect of *B. velezensis* SAAS-63 inoculation on the accumulation of nutrients in the plants under different nutrient conditions, the contents of macronutrients and micronutrients were measured. The accumulation of macronutrients in the plants showed a similar pattern under nutrient-deficient conditions was that macronutrients accumulated in large quantities in the roots but at low levels in the leaves (Fig. 3a–c). By contrast, under nutrient-sufficient conditions, inoculation with *B. velezensis* SAAS-63 caused the accumulation of macronutrients in the roots to be significantly reduced compared with nutrient-deficient conditions, whereas macronutrient accumulation significantly increased in the leaves. Under nutrient-deficient conditions and inoculation with *B. velezensis* SAAS-63, the contents of N, P, and K in the roots decreased by 89.11%, 34.58%, and 49.56%, respectively, while the contents of N, P, and K in the leaves increased by 120.31%, 19.75%, and 10.47%, respectively. The accumulation of micronutrients also changed after inoculation with *B. velezensis* SAAS-63 (Fig. 3d–f). Under nutrient-deficient conditions, the contents of Ca, Mn, and Zn in the roots inoculated with *B. velezensis* SAAS-63 decreased by 58.35%, 89.09%, and 19.91%, respectively, and the contents of Ca, Mn, Zn, and Cu in the leaves increased by 27.50%, 120.12%, and 13.79%, respectively. In contrast to the macronutrients, the accumulation of micronutrients in the roots increased following inoculation with *B. velezensis* SAAS-63 under nutrient-sufficient conditions, which did not affect the accumulation of micronutrients in the leaves, and these values were still significantly higher than in the non-inoculation treatment.

### Changes in microbial community composition and diversity following *B. velezensis* SAAS-63 inoculation

**Fig. 4:**
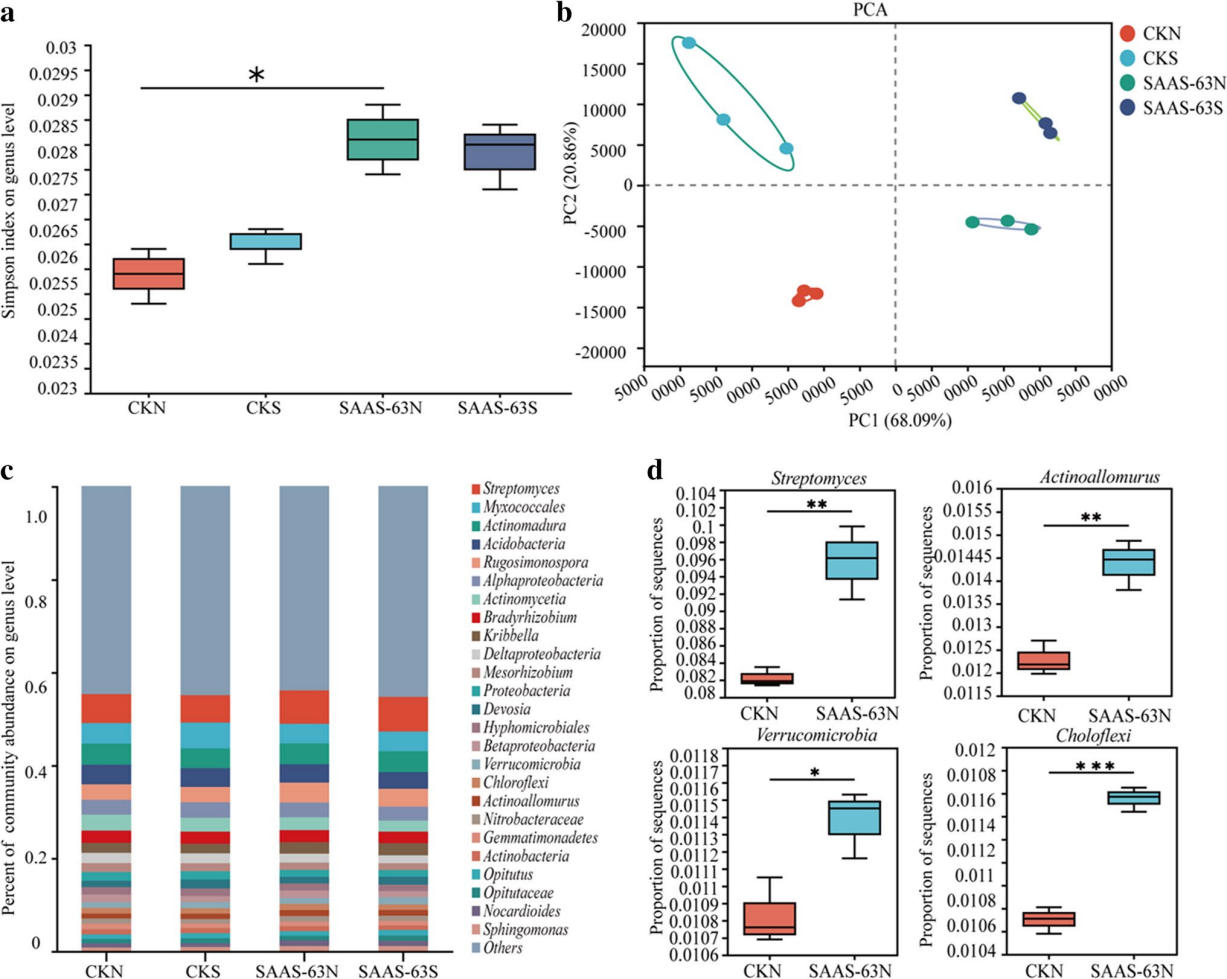
Comparative metagenomic analysis of plants inoculated with *B. velezensis* SAAS-63 or not under different nutrient conditions. **a** Differences in the microbial Simpson index in the rhizosphere soil under different treatments. Black asterisks indicate the significance of α-diversity index in soils (^*^p < 0.05; Wilcoxon rank-sum test). **b** PCA of rhizosphere soil sample community differences based on Bray–Curtis distances among metagenomic profiles. **c** Relative abundance in the rhizosphere microbial community at the genus level. **d** Differences in beneficial bacteria in the rhizosphere among different treatment groups (^*^p < 0.05, ^**^p < 0.01, and.^***^p < 0.001; Wilcoxon rank-sum test)

In this study, the diversity of soil microorganisms was analyzed. The Simpson index of CKN was the lowest. Inoculation with strain SAAS-63 significantly increased the Simpson index of the rhizosphere soil microorganisms under nutrient deficiency (Fig. 4a). The results showed that inoculation with *B. velezensis* SAAS-63 increased soil microbial diversity. The PCA based on the microbial taxonomic level (genus) and functional classification showed that different groups of treatments under different nutrient conditions formed different clusters (Fig. 4b). The results showed that different nutrient conditions and inoculation with *B. velezensis* SAAS-63 influenced the composition and function of the microbial community in the plant rhizosphere. According to the analysis of the relative abundance of species at the genus level, the community structure of the rhizosphere microorganisms also changed (Fig. 4c). To determine the effect of *B. velezensis* SAAS-63 inoculation under nutrient-deficient conditions, microorganisms with a significant increase in relative abundance following *B. velezensis* SAAS-63 inoculation were searched, and the relative abundance of four beneficial microbial genera increased significantly, suggesting that these microorganisms help plants cope with nutrient deficiency (Fig. 4d). Under nutrient-deficient conditions, *B. velezensi*s SAAS-63 inoculation significantly increased the abundance of the beneficial taxa *Streptomyces*, *Actinoallomurus*, *Verrucomicrobia*, and *Chloroflexi*.

### Metabolic differences in rhizosphere soil across *B. velezensis* SAAS-63 treatments

**Fig. 5:**
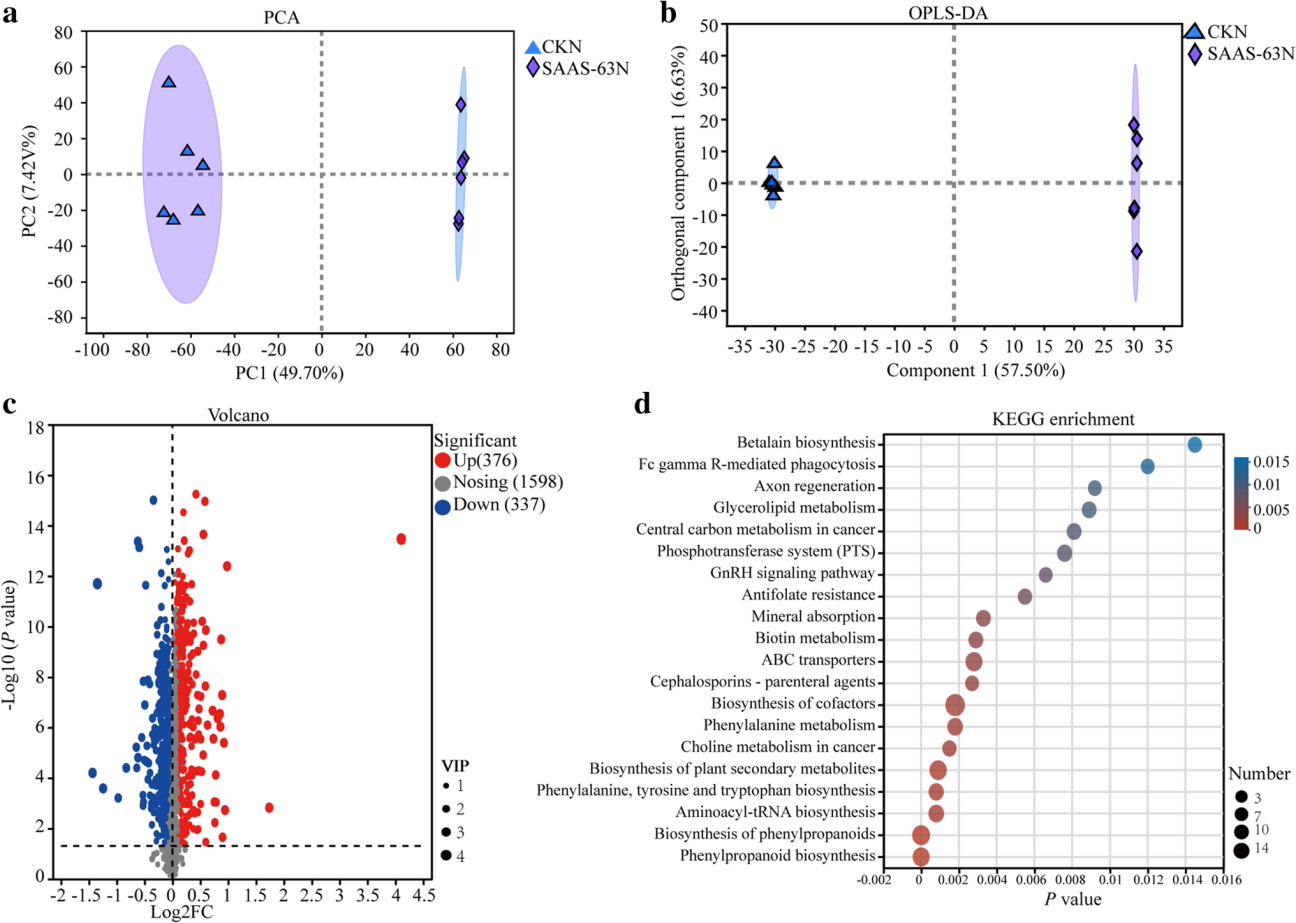
Comparative metabolomic analysis of plants inoculated with *B. velezensis* SAAS-63 or not under nutrient-deficient conditions. **a** PCA of rhizosphere metabolomic profiles based on Bray–Curtis distances. **b** OPLS-DA of rhizosphere metabolomic profiles. **c** Relative abundances of different metabolites. The volcano plot was obtained by plotting the log2fold change of the metabolites on the *x*-axis and the − log10 (*p* value) on the *y*-axis. VIP (variable importance in the projection) is the importance of variables to the model. Each point in the figure represents a specific metabolite, and the size of the point represents the VIP value. **d** The top 20 pathways of significantly upregulated and downregulated DAMs based on KEGG. The bubble size represents the number of DAMs involved. The bubble color indicates the enrichment degree of the pathway

The changes in metabolites in the rhizosphere soil were studied using nontargeted metabonomics. Inoculation with *B. velezensis* SAAS-63 caused changes in metabolites in the rhizosphere soil. The PCA of the metabolite spectrum showed that the first principal component and the second spectrum component significantly separated the metabolic profiles of the two treatment groups under nutrient-deficient conditions. PC1 and PC2 explained more than 57% of the variability and were mainly distinguished by PC1 (Fig. 5a). An OPLS-DA was performed, and the score showed that different treatments also displayed significant segregation (Fig. 5b). Comparing the changes in the metabolites in the two groups under nutrient deficiency, 376 metabolites were significantly upregulated and 337 metabolites were significantly downregulated under inoculation with *B. velezensis* SAAS-63 compared with the CK (Fig. 5c). A KEGG enrichment analysis was performed in the two treatment groups under nutrient-deficient conditions. The pathways of significant enrichment of the top four metabolites in the two treatment groups were compared, and three pathways related to phenylpropane metabolism were found (Fig. 5d). Therefore, we paid greater attention to the effect of *B. velezensis* SAAS-63 inoculation on the phenylpropanoid biosynthesis pathway in subsequent analyses.

**Fig. 6:**
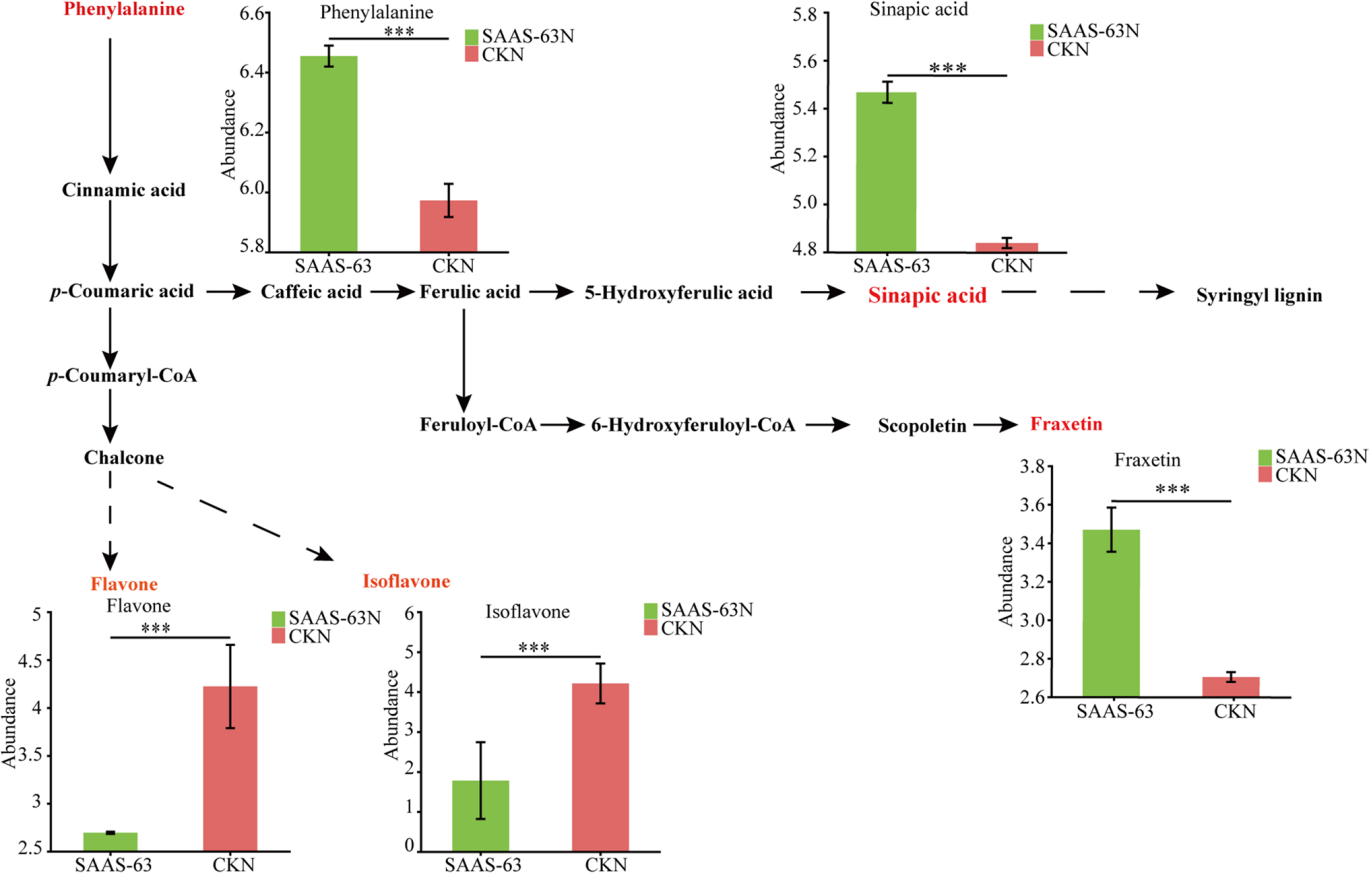
Changes in metabolites of the metabolic pathways in the plant rhizosphere subjected to nutrient-sufficient and nutrient-deficient conditions and inoculated with *B. velezensis* SAAS-63

Phenylpropanoid metabolism is one of the most important secondary metabolic pathways in plants. As the results of the KEGG enrichment analysis indicated significantly enriched phenylpropanoid metabolism, we analyzed the metabolites of this pathway (Fig. 6). The abundance of metabolites from different branches of this pathway changed after inoculation with *B. velezensis* SAAS-63 under nutrient deficiency. Phenylpropanoid metabolism starts with phenylalanine. At the beginning of phenylpropanoid metabolism, the abundance of phenylalanine increased significantly after *B. velezensis* SAAS-63 inoculation. As a substrate, the increase in the abundance of phenylalanine increased the abundance of metabolites in different metabolic branches. The abundance of sinapic acid and fraxetin, two metabolites from the phenylpropanoid metabolic branch, also increased after inoculation with *B. velezensis* SAAS-63. On the contrary, metabolites (including flavone and isoflavone) of another branch of phenylpropanoid metabolism were significantly decreased, suggesting the redirection of metabolic flux between the branches of the phenylpropanoid pathway.

**Fig. 7:**
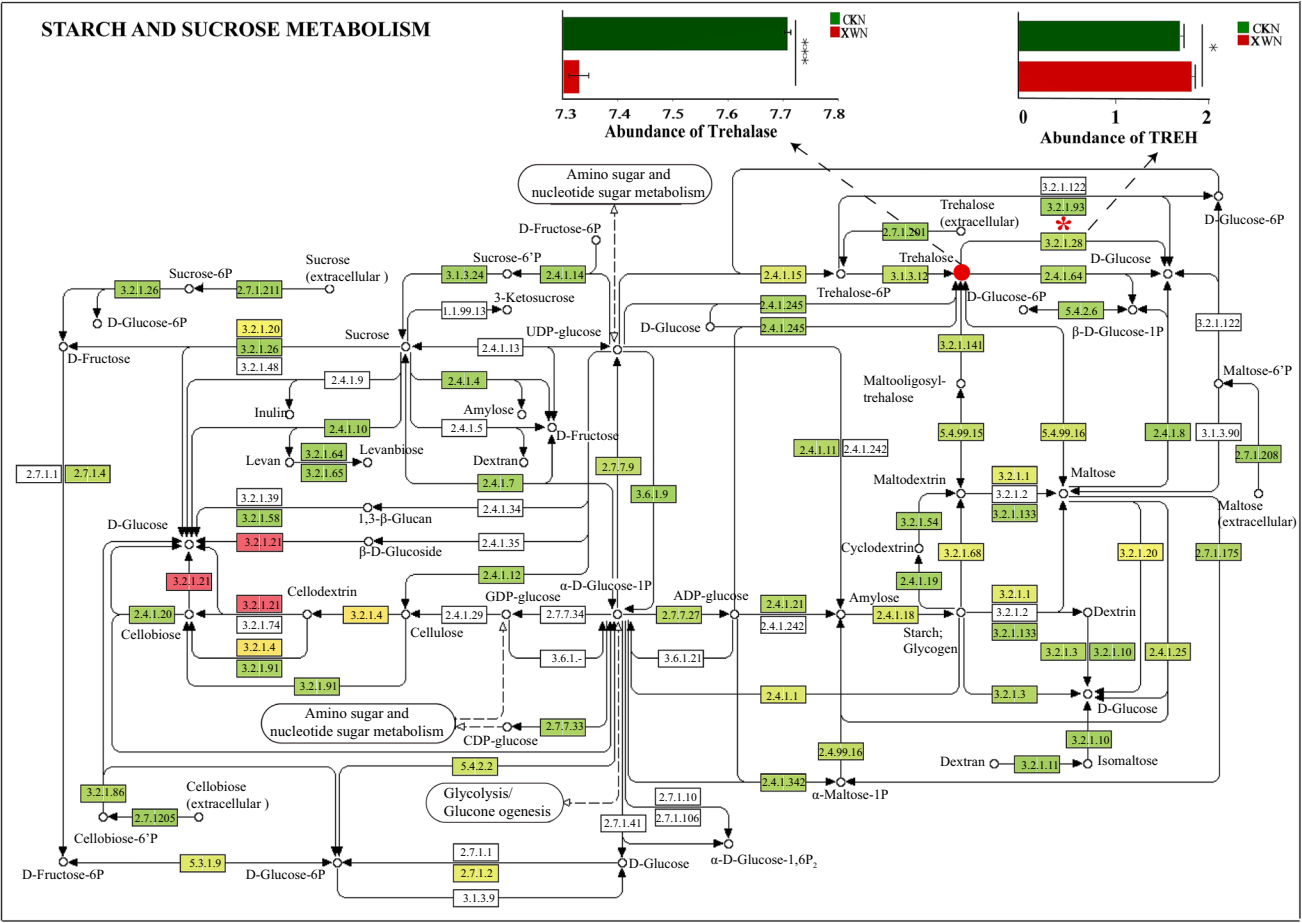
Starch and sugar metabolism pathways and associated significantly upregulated enzymes. CKN represents the control group without inoculation under nutrient-deficient conditions. SAAS-63N represents the group that was inoculated with *B. velezensis* SAAS-63 under nutrient-deficient conditions. TREH is the gene encoding trehalose hydrolase (EC3.2.1.28)

The KEGG pathways associated with representative metabolites were primarily involved in lipid metabolism, carbohydrate metabolism, and energy metabolism, among other areas (Supplemental Fig. S1). Combined “omics” methods revealed that differentially enriched KEGG pathways in the SAAS-63N metagenomes, compared to the CKN metagenomes, included starch and sucrose metabolism (ko00500), which is a component of carbohydrate metabolism (Fig. 7). After inoculation with *B. velezensis* SAAS-63, the abundance of many genes encoding starch and sucrose metabolic pathway enzymes changed (Supplemental Fig. S2). The abundance of the gene *TREH* encoding trehalose hydrolase (EC3.2.1.28) in the SAAS-63N metagenome was significantly higher than that in the CKN metagenome (Fig. 7). In addition, we searched for the associated metabolites of the enzymes whose gene abundances were altered and found that the trehalose content decreased significantly after inoculation with strain SAAS-63. The results showed that strain SAAS-63 inoculation could recruit specific rhizosphere microorganisms to use trehalose to re-establish the pathway of carbon (C) metabolism in the rhizosphere.

### Relationship between metabolites and rhizosphere soil microorganisms

**Fig. 8:**
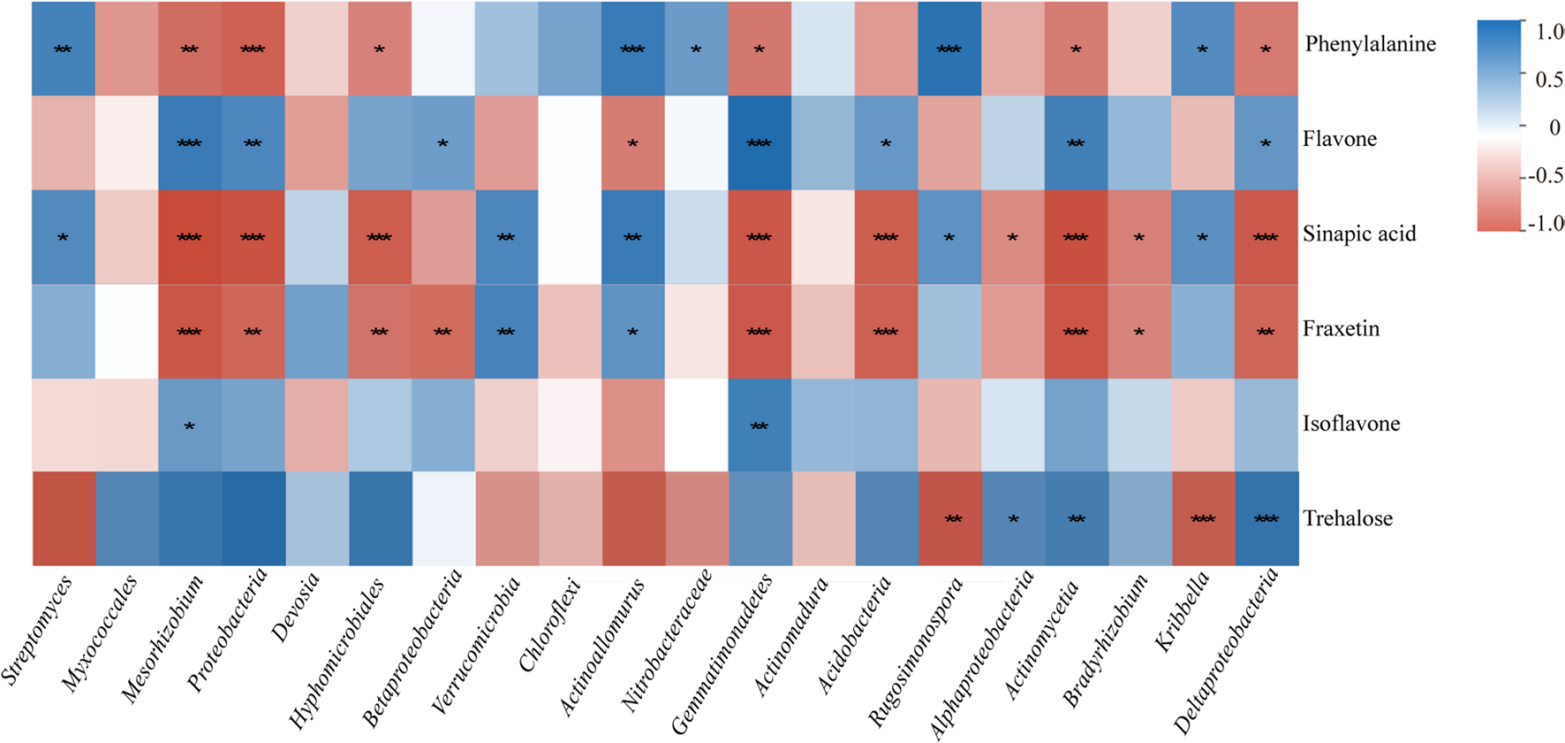
Correlations of the top 20 microorganisms in relative abundance with six differential metabolites. *p < 0.05, **p < 0.01, and ***p < 0.001, respectively

Explaining the relationship between microbes and metabolites is a necessary step in exploring how PGPRs improve plant resistance to nutrient deficiency. Therefore, we searched for correlations between the top 10 genera and obtained DAMs. Significant (*p* < 0.05) and highly significant (*p* < 0.01) correlations were observed between certain rhizosphere microbes and differential metabolites (Fig. 8). Phenylalanine correlated significantly with *Actinoallomurus* (*r* = 0.8287, *p* < 0.001) and *Rugosimonospora* (*r* = 0.9041, *p* < 0.001). Flavone was highly positively correlated with *Mesorhizobium* (*r* = 0.8233, *p* < 0.001) and *Gemmatimonadetes* (*r* = 0.9571, *p* < 0.001). Sinapic acid was positively correlated with *Verrucomicrobia* (*r* = 0.7282, *p* < 0.01) and *Actinoallomurus* (*r* = 0.8229, *p* < 0.01). Fraxetin was positively correlated with *Verrucomicrobia* (*r* = 0.7632, *p* < 0.01) and *Actinoallomurus* (*r* = 0.6477, *p* < 0.05). It is worth noting that trehalose had a significant positive correlation with most rhizosphere microorganisms. Meanwhile, trehalose showed a highly positive correlation with *Mesorhizobium* (*r* = 0.8326, *p* < 0.001), *Proteobacteria* (*r* = 0.9106, *p* < 0.001), *Hyphomicrobiales* (*r* = 0.8335, *p* < 0.001), and *Deltaproteobacteria* (*r* = 0.8388, *p* < 0.001). This may explain the significant decrease in trehalose in the rhizosphere following *B. velezensis* SAAS-63 inoculation. The results showed that the relative abundance of six microorganisms had the strongest correlation with certain metabolites in the rhizosphere, indicating that these microorganisms may be involved in the formation of most of the metabolites in the soil.

## Discussion

As an abiotic stress, nutrient stress seriously inhibits plant growth. As beneficial microorganisms, PGPRs have great potential to not only promote plant growth but also to help plants resist abiotic stress (Karnwal et al. 2023). At present, many studies have reported the ability of PGPR to promote growth and resist abiotic stress. However, most studies have focused on the antioxidant capacity and osmotic regulation of PGPR under drought, salinity, and high temperature stress. The understanding of the changes of rhizosphere microorganisms and metabolites of plant-PGPR interaction under nutrient stress is quite limited. In the post-genome era, metagenomics and metabonomics are considered to be important tools for modern agriculture to reveal complex mechanisms through microorganisms and metabolites. Therefore, this study not only systematically studied the effects of PGPR on the physiology and biochemistry of lettuce but also explored the nutrient stress of PGPR from the gene and molecular level. In this study, inoculation with strain SAAS-63 promoted the growth of lettuce under nutrient-sufficient and nutrient-deficient conditions and improved agronomic parameters. Notably, the growth of seedlings inoculated with strain SAAS-63 under nutrient-deficient conditions was most improved. Some studies have also reported increased growth under abiotic stress following the application of PGPRs (Nigam et al. 2022). In this study, the root length under nutritional stress was shorter than that under adequate nutrition. The root length increased significantly after inoculation with *B. velezensis* SAAS-63, which indicated that inoculation with PGPRs promoted plant root development and helped the plants to absorb more surrounding nutrients.

Abiotic stress destroys the osmotic balance and induces the excessive production of ROS in plant cells. Enzymatic antioxidant systems and nonenzymatic components play key roles in the induction and elimination of toxic levels of ROS (Liebthal et al. 2018). In this experiment, the activity of SOD, POD, and CAT in the inoculated plants leaves was significantly higher than that of the non-inoculated plants. This phenomenon showed that the ability of scavenging reactive oxygen species of inoculated plants is higher than that of uninoculated plants under the nutrient-deficient conditions. In this study, the content of Pro increased significantly after inoculation with *B. velezensis* SAAS-63. Pro plays a variety of key roles, such as stabilizing cell membrane and protein, transmitting cell signals, and regulating gene expression (Muhammad et al. 2019). As an osmotic regulator, an increase in Pro content helps alleviate stress in plants (Guan et al. 2023). In addition, the improvement of nutrient stress resistance in the plants via inoculation with *B. velezensis* SAAS-63 did not cause an increase in the content of all osmotic regulating substances. For example, the content of SS decreased significantly after *B. velezensis* SAAS-63 inoculation. We speculated that inoculation with PGPRs promoted the decomposition and utilization of SS. In addition, the content of MDA in the plants increased significantly under nutrient deficiency and decreased significantly after inoculation with *B. velezensis* SAAS-63. MDA is produced by plasma membrane lipid peroxidation (LPO) (Rashid et al. 2021). As one of the index of LPO, MDA can directly reflect the damage degree of stress to plants (Ahmad et al. 2016; Hasanuzzaman et al. 2011). When the MDA content is lower, the damage of MDA to plants is lower. The results indicated that inoculation with *B. velezensis* SAAS-63 helped to reduce the harm caused by nutrient stress to the plants. Thus, PGPRs can regulate the antioxidant capacity of plants through a series of biochemical reactions (enzyme system and non-enzyme system) to prevent oxidative damage caused by ROS.

Plant growth was limited under nutrient deficiency, which was consistent with the significant decrease in the accumulation of nutrients in the plant leaves. A higher concentration of macronutrients and micronutrients in the seedling leaves inoculated with *B. velezensis* SAAS-63 suggests the involvement of PGPRs in increasing N and mineral uptake (He et al. 2019; Nguyen et al. 2018). In addition, the accumulation of N and mineral elements in the roots of seedlings without *B. velezensis* SAAS-63 inoculation increased significantly, while that of the seedlings inoculated with *B. velezensis* SAAS-63 decreased significantly. The results showed that PGPR inoculation contributed to the transport of nutrients from the roots to the leaves. However, the role of PGPRs was not obvious under nutrient-sufficient conditions. Combined with the plant morphological index, the root length of the plants was significantly promoted by *B. velezensis* SAAS-63 inoculation. An increase in root length helps plants to absorb more nutrients. In addition, the positive role of PGPRs may also be related to some substances that promote plant nutrient absorption, such as melatonin and dopamine, which can promote plant mineral absorption under abiotic stress (Li et al. 2016). The effects of some mineral elements (Zn, Fe, and P) on transcriptional response, nutrient sensing, signal transduction, and transport in plant nutrition regulation also have been noted (Fan et al. 2021). Therefore, this study confirmed that PGPRs can change the sensing and signaling involved in plant nutrition to promote plant growth under the nutrient-deficient condition and this change is achieved by regulating the content of nutrient elements.

With changes in climate, crops are increasingly being subjected to abiotic stresses during cultivation. By settling in the rhizosphere of plants, PGPRs protect plants from many abiotic stresses. In this experiment, inoculation with *B. velezensis* SAAS-63 under nutrient deficiency significantly changed the diversity of plant rhizosphere microorganisms, which may be due to the interaction between *B. velezensis* SAAS-63 and native microorganisms to form a unique microbial network. The formation of soil microbial complex community can regulate the cycle of soil nutrients and affect soil properties, promote plant growth, and promote the sustainability of the ecosystem (Zhang et al. 2019). There were significant differences in multiple analyses between groups inoculated with *B. velezensis* SAAS-63 and those without inoculation, indicating that inoculation with PGPRs influenced the rhizosphere microbial community structure. Under nutrient deficiency, *B. velezensis* SAAS-63 inoculation significantly increased the abundance of *Streptomyces*, *Actinoallomurus*, *Verrucomicrobia*, and *Chloroflexi*. *Streptomyces* can relieve the pressure caused by the environment and increase the yield of plants. A previous study demonstrated that *Actinoallomurus* is related to the synthesis of flavonoids and coumarins, which is beneficial to the synthesis of substances resistant to environmental stress (Pozzi et al. 2011). In the correlation analysis between the rhizosphere microorganisms and metabolites, we also found a significant correlation between C and the coumarin precursor sinapic acid, which shows that *Actinoallomurus* plays an important role in the production of stress-resistant substances in plants. *Verrucomicrobia* plays a vital role in agricultural sustainability and the promotion of plant and crop yield by availing nutrients and preparing a conducive environment (Baliyarsingh et al. 2022). Research shows that *Chloroflexi* are key bacteria that help improve resistance (Wang et al. 2021). Therefore, this study showed that the structure of rhizosphere microorganisms was altered by inoculating plants with *B. velezensis* SAAS-63, with more beneficial bacteria recruited to improve their ability to resist nutritional deficiency.

To explore the effect of strain SAAS-63 inoculation on improving plant response to nutritional stress, the metabolites in the rhizosphere were also analyzed. There are two main sources of soil metabolites, one from plant roots and the other from microorganisms. The composition of root exudates varies with plant species and environmental stress (Cheng et al. 2022). A former study have shown that exogenous inoculants can alter plant metabolism (Su et al. 2023). Our study found that inoculation with *B. velezensis* SAAS-63 under nutrient deficiency significantly affected the soil metabolite spectrum, including organic acids and derivatives and lipids and lipid-like molecules. It also interfered with some metabolic pathways including those involved in basic C metabolism and some secondary metabolic pathways. Firstly, the metabolic pathway of phenylpropanoid changed significantly after *B. velezensis* SAAS-63 inoculation in this study. When the plants were subjected to nutritional stress, the contents of flavonoids and isoflavones in the non-inoculated plants were significantly higher than those in the inoculated plants. Flavonoids and isoflavones are considered important substances for plants to resist abiotic stress (Pozzi et al. 2011; Trush and Pal'ove-Balang 2023). Therefore, the decrease of these substances after inoculation with *B. velezensis* SAAS-63 indicated that *B. velezensis* SAAS-63 can help plants to resist stress. Secondly, to further explore the mechanism of resistance of *B. velezensis* SAAS-63 to stress, we analyzed other substances in the phenylpropanoid metabolic pathway and found that the contents of sinapic acid and fraxetin increased significantly after *B. velezensis* SAAS-63 inoculation. Sinapic acid is the precursor of lignin, and an increase in sinapic acid content helps in plant lignin synthesis. Lignin is a major player for plants to perceive and respond to environmental stress. It can not only provide mechanical strength for plant secondary cell wall but also protect cells from abiotic stress (Jia et al. 2019). Fraxetin, which is a coumarin, also plays an important role in reducing abiotic stress in plants (Singh et al. 2021). A former study showed that plants can release coumarins to the rhizosphere under abiotic stress, which is a key means by which plants can obtain iron (Chutia et al. 2019). As indicated in Fig. 7, the synthesis of flavonoids and isoflavone decreased, and more phenylpropane flowed to the lignin synthesis pathway and coumarin synthesis pathway. Therefore, the redirected metabolic flux after *B. velezensis* SAAS-63 inoculation explained the mechanism by which *B. velezensis* SAAS-63 helps plants to resist nutrient stress. Plants need to constantly adjust the distribution of energy and metabolites to maintain growth and survive under stress (Zhang et al. 2020). In addition, the primary metabolism of the plants also changed after inoculation with *B. velezensis* SAAS-63. Carbon metabolism includes starch and sucrose metabolism, glycolysis, and the tricarboxylic acid cycle (Hartman et al. 2023). Trehalose metabolism is a branch of starch and sucrose metabolism. A former study showed that trehalose plays an important role in plant growth and the stress response and can promote root growth and stomatal closure (Kosar et al. 2019). When the trehalose content decreased after inoculation with *B. velezensis* SAAS-63, the abundance of genes encoding trehalose hydrolases in the plant rhizosphere microbial macrogenomes increased, which indicated that *B. velezensis* SAAS63 inoculation could help plants recruit microorganisms to decompose and utilize trehalose. Research has indicated that starch and sucrose metabolism function are significantly correlated with different bacteria along different metabolite pathways, which may confirm that different kinds of bacteria lead to different metabolisms by using and decomposing starch and sucrose (Song et al. 2020). Meanwhile, the phenylpropane metabolic pathway is also induced by C metabolism (Zhang et al. 2018). To sum up, the addition of PGPRs can alter the metabolism of the plant rhizosphere. These results suggested that PGPRs can improve the stress tolerance of plants by affecting primary and secondary metabolism.

Soil is a quasi-organism, in which microorganisms undertake most of the metabolic activities of soil. The changes of soil metabolites often depend on the species and abundance of soil microorganisms. Complex microbial communities play a decisive role in the cycle and metabolism of exogenous nutrients in soil. Therefore, it is a meaningful work to analyze and reveal the correlation between soil metabolism and bacterial community. The results of our analysis showed that the changes in several metabolites following *B. velezensis* SAAS-63 inoculation were closely related to microorganisms. A former study showed that flavonoid derivatives secreted by the roots drive oxalic acid bacteria to become enriched in the rhizosphere, which in turn promotes lateral root development and N uptake in maize in low N soil (Yu et al. 2021). In this study, the differential metabolites were significantly enriched in the phenylpropane metabolic pathway, which is an important branch of N metabolism. This shows that rhizosphere microorganisms play an essential role in N metabolism. Song et al. (2020) showed that the metabolic of starch and sucrose were most closely related to different bacterial members in different metabolites pathways, which may confirm that bacteria regulate starch and sucrose metabolic and lead to different types of metabolism in the rhizosphere. This further confirms that the decrease in trehalose abundance in the starch and sucrose metabolic pathways in this study was due to the decomposition of specific microorganisms in the rhizosphere and the use of trehalose to rebuild rhizosphere C metabolism. Our results show that the changes in several metabolites induced by *B. velezensis* SAAS-63 inoculation are related to the changes in bacterial and fungal communities. This result further supports the view that soil microorganisms can promote or inhibit the accumulation of soil metabolites. Based on metagenomics and metabolomics, this study comprehensively analyzed the changes in rhizosphere microorganisms and metabolites after inoculation with *B. velezensis* SAAS-63, identifying key metabolic pathways. However, these results do not fully explain the exact mechanism by which PGPRs help plants to resist nutritional stress. Further work needs to be done to analyze the mechanisms of PGPR resistance to stress. Transcriptomic analysis and dynamic monitoring of the changes in metabolites and related microorganisms are necessary to further reveal the mechanism by which inoculation with PGPRs improves resistance to nutritional stress. Our results confirm the close relationship between soil microorganisms and soil metabolism, which helps us understand the mechanism of tolerance of lettuce to nutrient deficiency induced by PGPRs. This can guide the measures for improving soil quality and crop yield via the addition of PGPRs, thereby providing an important reference for the further study of plant–microorganism–environment interactions.

## Supplementary Information

Below is the link to the electronic supplementary material.

Supplementary file1 (PDF 638 KB)

## Data Availability

The raw data supporting the conclusions of this article will be made available by the authors, without undue reservation, to any qualified researcher. The raw reads were deposited into the NCBI Sequence Read Archive (SRA) database (accession codes PRJNA1039622).
