# Supplementary material for: Using multi-omics to explore the effect of Bacillus velezensis SAAS-63 on resisting nutrient stress in lettuce
Source: Appl Microbiol Biotechnol. 2024 Apr 29;108(1):313. doi: 10.1007/s00253-024-13153-y (PMC11058974; doi:10.1007/s00253-024-13153-y)
Supplement: Supplementary file 1 — Supplementary file1 (PDF 638 KB) [file 253_2024_13153_MOESM1_ESM.pdf]

Journal name: Applied Microbiology and Biotechnology

Manuscript Title: Using multi-omics to explore the effect of *Bacillus velezensis* SAAS-63 on resisting nutrient stress in lettuce

Yinshuang Bai [1,2,#], Ke Song [2,#], Mengxiang Gao[1], Juan Ma[4], Yifan Zhou[2], Hua Liu[2,3], Haijuan Zeng[2,3], Jinbin Wang[1,2,3,*] , Xianqing Zheng[2,*]

[1] *College of Life Sciences, Yangtze University, Jingzhou 434025, China*

[2]*The Biotechnology Research Institute, The Eco-Environmental Protection Research Institute, Shanghai Academy of Agricultural Sciences, Key Laboratory of Agricultural Genetics and Breeding, Shanghai 201106, China*

[3]*Crops Ecological Environment Security Inspection and Supervision Center, Key Laboratory for Safety Assessment of Agricultural Genetically Modified Organisms, Ministry of Agriculture and Rural Affairs, Shanghai 201106, China*

[4]*School of Life Science and Engineering, Lanzhou University of Technology, Lanzhou 730050, China*

* Correspondence: Corresponding Author. E-mail: wangjinbin2013@126.com (J. Wang), zhengxianqing@saas.sh.cn (X. Zheng)

[#] *Authors contributed equally to the manuscript.*

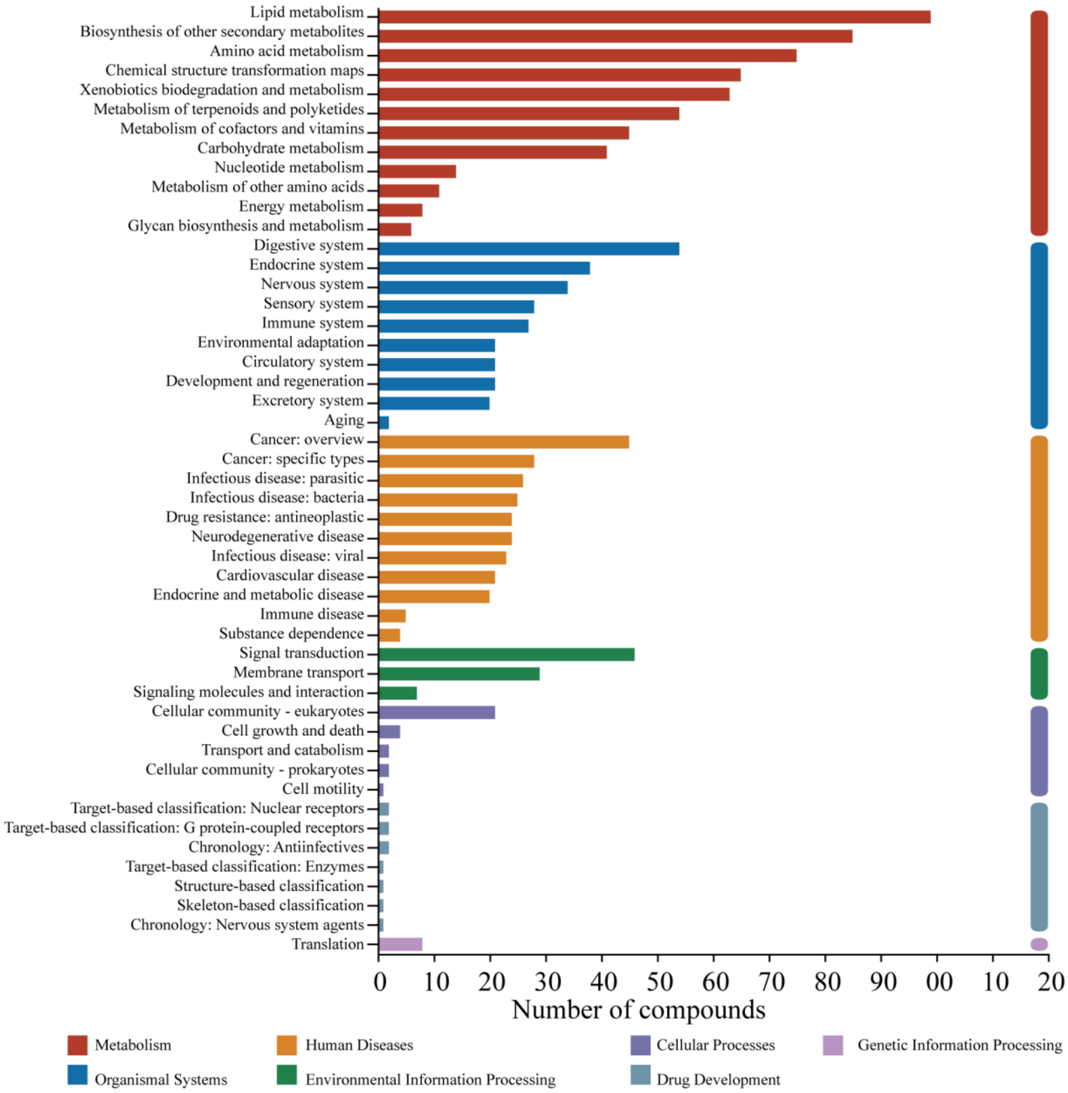


Fig. S1 KEGG pathways. The ordinate is the secondary classification of KEGG metabolic pathway, and the abscissa is the number of compounds annotated to this pathway. KEGG metabolic pathways can be divided into seven categories: metabolism,genetic information processing, environmental information processing, cellular processes, organismal systems, human diseases, drug development. The colors of the bars indicate different metabolic pathway classes.

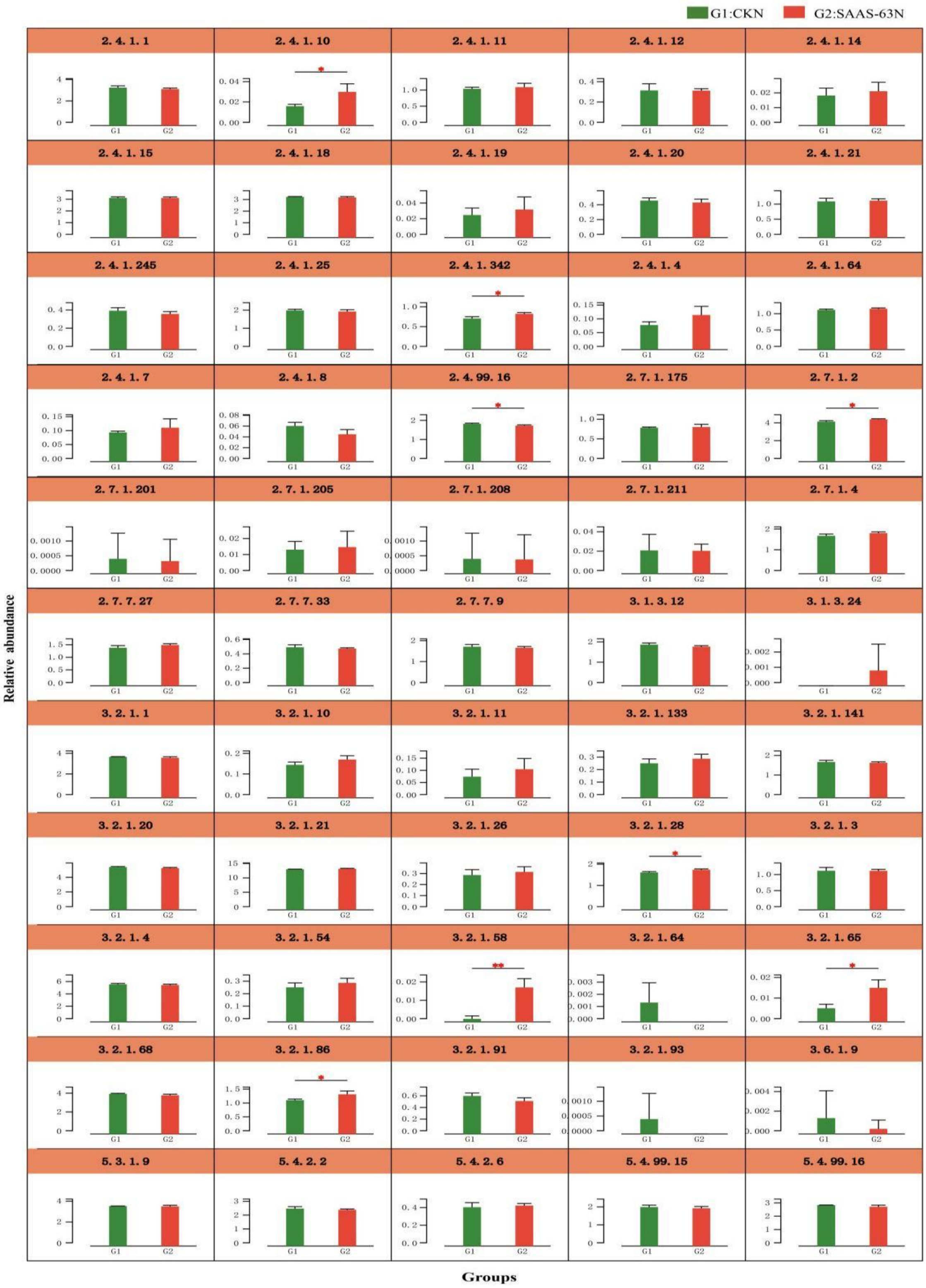


Fig. S2 Abundance of genes encoding-related enzymes during starch and sucrose metabolism in the two treatments. CKN represents the control group without inoculation under nutrient-deficient conditions. SAAS-63N represents the group that was inoculated with *B. velezensis* SAAS-63 under nutrient-deficient conditions. The numbers in the pink bars on to of each subfigure represents different enzymes in a metabolic pathway.
